# A Facile Synthesis Route of Hybrid Polyurea-Polyurethane-MWCNTs Nanocomposite Coatings for Ballistic Protection and Experimental Testing in Dynamic Regime

**DOI:** 10.3390/polym13101618

**Published:** 2021-05-17

**Authors:** Gabriela Toader, Aurel Diacon, Edina Rusen, Florica Rizea, Mircea Teodorescu, Paul O. Stanescu, Celina Damian, Adrian Rotariu, Eugen Trana, Florina Bucur, Raluca Ginghina

**Affiliations:** 1 Faculty of Weapon Systems Engineering and Mechatronics, Military Technical Academy, 39-49 George Cosbuc Boulevard, 050141 Bucharest, Romania; nitagabriela.t@gmail.com (G.T.); adrian.rotariu@mta.ro (A.R.); eugen.trana@mta.ro (E.T.); florina.bucur@mta.ro (F.B.); 2 Department of Bioresources and Polymer Science, Faculty of Applied Chemistry and Materials Science, University Politehnica of Bucharest, 1-7 Gh. Polizu Street, 011061 Bucharest, Romania; edina.rusen@upb.ro (E.R.); flori_rizea@yahoo.com (F.R.); mircea.teodorescu@upb.ro (M.T.); paul_stanescu@yahoo.com (P.O.S.); celina.damian@yahoo.com (C.D.); 3 Scientific Research Center for CBRN Defense and Ecology, 225 Soseaua Oltenitei, 041327 Bucharest, Romania; ginghinaraluca@gmail.com

**Keywords:** polyurea-polyurethane, nanocomposite, ballistic protection, coatings, mechanical properties, multiwall carbon nanotubes (MWCNTs), Hopkinson bar

## Abstract

This study describes a simple, practical, inexpensive, improved, and efficient novel method for obtaining polyurea-polyurethane-multiwall carbon nanotubes (MWCNTs) nanocomposites with enhanced mechanical properties, and their experimental testing in a dynamic regime. SEM and micro-CT investigations validated the homogeneity of the nanocomposite films and uniform dispersion of the nanofiller inside the polymeric matrix. The experimental measurements (TGA, DSC, DMA, and tensile tests) revealed improved thermal and mechanical properties of these new materials. To demonstrate that these nanocomposites are suitable for ballistic protection, impact tests were performed on aluminum plates coated with the polyurea-polyurethane MWCNTs nanocomposites, using a Hopkinson bar set-up. The experimental testing in the dynamic regime of the polyurea- polyurethane-coated aluminum plates confirmed that the nanocomposite layers allow the metal plate to maintain its integrity at a maximum force value that is almost 200% higher than for the uncoated metallic specimens.

## 1. Introduction

Nowadays, war migrates from the battlefields towards less predictable areas. All existing ammunition types and improvised explosive devices represent serious threats. The survival of defense, public order, or national security crews, and the vehicles that they use during a mission, is conditioned by several factors, including ballistic protection. Specific categories of threat require different levels of ballistic protection, regulated by the standards valid in each state.

New effective solutions for ballistic protection are offered by the evolution of composite materials, the synthesis of new materials, and the selection and correlation of performant materials [1]. These materials must be resistant to the action of a shock wave or the impact of a projectile [2,3,4,5], but they must also be lightweight so they do not overload the armor of a trooper or vehicle [6]. Composites reinforced with aramid fibers are described in the literature as one of the materials with the highest ratios between weight (mass) and level of impact protection, but their cost is considered a disadvantage [6,7]. Another alternative to steel armor is the use of an aluminum substrate coated with composite materials [8]. Numerous studies have shown that the rigidity and hardness of a material can be improved using nanoparticles [7,9,10,11,12].

To obtain superior mechanical properties for a particular type of polymeric matrix, it is necessary to take into account the size, shape, and chemical properties of the nanometric filler particles [13]. Multiwall carbon nanotubes (MWCNTs) have been extensively used in nanocomposites due to their outstanding properties: high Young’s modulus, stiffness, flexibility, and conductivity. Additionally, only a 1% MWCNTs content can lead to an increase of up to 36–42% of the modulus of elasticity for composite material [14,15,16]. Even though carbon nanotubes have great potential as nanofillers, a major concern related to the performance of MWCNTs-based nanocomposites is represented by the difficulty of achieving their homogenous dispersion. The dispersability of carbon nanotubes in the polymer matrix is conditioned by their chemical and physical compatibility. Therefore, in some cases, the functionalization of MWCNTs can become mandatory for their compatibilization with the organic substrate.

Polyurea has been widely reported as being an appropriate polymeric matrix for impulsive loading ballistic protection [17,18,19,20,21,22], due to its embedded reinforcing nanoscale hard domains, uniformly dispersed and chemically linked inside its soft elastic nanodomains [9,23]. Therefore, many researchers have investigated polyurea for their distinctive synergistic properties. Li et al. [24] employed Jeffamine^®^ D2000 and two different types of isocyanates, isophorone diisocyanate and hexamethylene diisocyanate, to obtain self-healing elastomers. Even though they possess this self-healing property, these elastomers exhibit insufficient mechanical resistance (maximum tensile stress of approximately 3.5 MPa) for impulsive loading applications. Therefore, numerous studies [17,25,26] confirmed the beneficial contribution of polyurea coatings applied to metallic surfaces. Bai et al. [27] obtained a polyurea with improved mechanical resistance by employing diphenylmethane diisocynante and Unilink 4200 diamine, reaching a maximum true stress of approximately 13 MPa (in the quasi-static regime, at low strain rates) and 27 MPa under a dynamic regime. Li et al. [28] investigated the response of stainless steel plates coated with a commercial polyurea (LINE XS-350) to impulsive loadings. This commercial polyurea displayed a maximum quasi-static true stress of only 22.4 MPa. The use of polyurea coatings affords a better response of the coated metal sheet at the action of a shock wave or the impact of a projectile by suffering lower deformations than the neat metallic plates. Therefore, in terms of ballistic protection, the efficacy of polyurea is already validated by an important volume of data available in the literature. Polyurea-MWCNTs systems [29,30,31] were also demonstrated in multiple studies as good candidates for impulsive loading applications. However, one major inconvenience is that the chemical modification of the nanofiller sometimes involves significant costs that may not prove to be economically viable.

In many cases, a good dispersion of nanoparticles in the polymer matrix can be obtained only by prior functionalization. This process can sometimes be difficult to perform and expensive, as the synthesis process involves several steps. To simplify the manufacturing process and to reduce the production costs for polyurea nanocomposites designed for ballistic protection, a commercially available MWCNT product was used. This product consists of a concentrate of MWCNTs pre-dispersed in a polyester-polyol system, which ensures a good dispersion in the polyurea matrix. This simplified synthesis method could be more advantageous for coating extended areas specific for ballistic protection applications. Thus, the metal substrates can be protected against cracking/failure by being coated with this high-performance polyurea nanocomposite by simply spraying the premixed reactants onto the targeted surface.

Therefore, taking into consideration the unique set of properties of polyurea-polyurethane and the multiple advantages of using pre-dispersed MWCNTs, this study intended to provide a novel approach towards a new facile synthesis route for obtaining high-performing polyurea-polyurethane nanocomposite coatings. This study also comprises a section on the experimental testing in a dynamic regime in order to prove the real contribution of this type of nanocomposite coating on the deformation mitigation of metallic plates for assessing the suitability of the materials’ design in relation to ballistic protection applications. To improve the response of the metallic structures to impulsive loadings, we herein decided to employ our previously synthesized polyurea matrix [17], which was obtained from poly (propylene glycol) bis(2-aminopropyl ether) with Mn ≈ 2000 Da, diphenylmethane-4,4′-diisocyanate, and 4′-diaminodiphenylmethane as chain extender. This polyurea possesses superior mechanical resistance, displaying a maximum quasi-static true stress of 33.76 MPa. Its unique set of properties established the premises for obtaining a performant nanocomposite with superior mechanical resistance (up to 40.84 MPa maximum quasi-static true stress) for ballistic protection applications. To the extent of our knowledge, this is the first paper that proposes an improved route for obtaining performant polyurea-polyurethane nanocomposites with pre-dispersed MWCNTs, conjoined with the evaluation of the mitigation effect brought by these performant materials and using the Hopkinson bar method. The novelty of this paper consists of both the straightforwardness of the polyurea coating fabrication method and the experimental set-up approach for the evaluation of the behavior of these materials at impact with a projectile. 

## 2. Materials and Methods

### 2.1. Materials

Poly (propylene glycol) bis(2-aminopropyl ether) − Mn ≈ 2000 Da (PPG2000, Sigma Aldrich, St. Louis, MO, USA), 4,4′-diaminodiphenylmethane (DADPHM, Sigma Aldrich, St. Louis, MO, USA), and diphenylmethane-4,4′-diisocyanate (MDI, technical product Desmodur^®^ 44V20L, Covestro, Leverkusen, Germany) were pre-dispersed in multiwalled carbon nanotubes in a polyester polyol based resin (MWCNTs, Graphistrength^®^ CPU2-30, Arkema, Colombes, France; Graphistrength^®^ CPU2-30 is a MultiWall Carbon Nanotubes (MWCNT), at a concentrate that is used as an additive for polyurethane-based materials, coatings or adhesives. It contained 30 wt% of MWCNT, perfectly dispersed in a polyester polyol. Typical final MWCNT loadings in the final compounds were in the range 0.1 to 2 wt%, depending on the host matrix characteristics, targeted performances, processing methods, and conditions. Acetone (Sigma Aldrich, St. Louis, MO, USA), was used as received.

### 2.2. Methods

#### 2.2.1. Preparation of Polyurea-Polyurethane Nanocomposite Films

Five distinct types of polyurea-polyurethane nanocomposites were synthesized (Table 1) to obtain polymeric films with different mechanical properties.

For the synthesis of the blank sample, the diamines (PPG2000 and DADPHM) and the isocyanate (MDI) were solubilized separately in acetone, obtaining two solutions: Solution **A** (containing the two components with amino functional groups, PPG2000: DADPHM molar ratio 1:2) and Solution **B** (containing the component with isocyanate functional groups). The molar ratio between the isocyanate and the amino (primary amine) groups was maintained for all reactions at 1:1. Solutions **A** and **B** were vigorously stirred for 10 to 15 s at room temperature, and then the mixture was transferred on a Petri dish placed on a perfectly horizontal surface. The reaction mixture was maintained at 20 °C and relative humidity of between 50% and 55%, until the reaction was complete. After 24 h, a polyurea film with a thickness of about 0.4 mm was obtained.

**Scheme 1:**
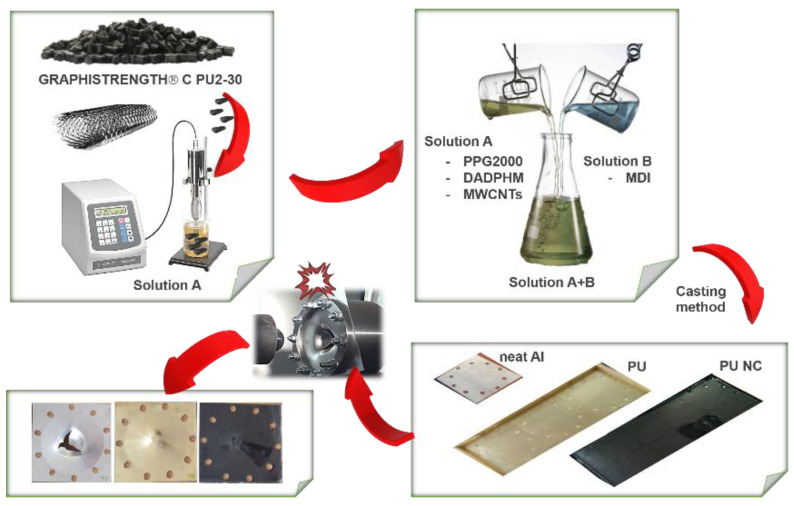
Representation of the fabrication steps for the polyurea-polyurethane nanocomposite films.

Polyurea-polyurethane-based composites were obtained using the same procedure, according to the compositions described in Table 1. The only difference was the introduction of carbon nanotubes in solution **A**, thus obtaining Solution **C**. To obtain the best possible dispersion of MWCNT in the polyurea matrix, solution **C** was subjected to the action of ultrasound for one hour, before being mixed with solution **B**. For a better understanding of the behavior of the synthesized polymeric films on impact with a projectile, samples consisting of aluminum plates coated with polyurea-polyurethane nanocomposites (described in Table 1) were prepared by a casting technique (Scheme 1).

The nanocomposites, obtained as described above, contain a hybrid polymeric matrix consisting of both polyurea and polyurethane zones. The polyurethane regions surround the MWCNTs, since these carbon nanotubes were pre-dispersed in a polyester polyol matrix before being part of the polyurea matrix. Even though these polyurethane areas are present in the nanocomposite, their concentration is neglectable in comparison with the polyurea matrix.

#### 2.2.2. Characterization

The morphology of the polyurea-polyurethane-MWCNTs nanocomposite films was investigated by SEM (scanning electron microscopy) using a Tescan Vega II LMU SEM instrument (TESCAN, Brno, Czechia) at 10 keV acceleration voltage. The distribution of the MWCNTs inside the polyurea-polyurethane matrix was examined via micro-CT technique. The SkyScan micro-CT attachment allowed for converting the Tescan Vega II LMU SEM to an X-ray microtomograph for non-destructive imaging and for measuring of the object’s internal microstructure of specimens. Analysis parameters: Exposure time—4 s per projection at electron beam currents of 100 nA; accelerating voltage—30 KeV; step size—1°; scanning time—24 min. Reconstruction was performed by the NRecon program which used float-point data values for internal calculations during reconstruction, and afterward allowed the operator to define the density window as a range of the reconstructed values. The full set of reconstruction results was visualized by the program DataViewer^®^ 2D/3D Micro-CT Slice Visualization (Micro Photonics Inc., Allentown, PA, USA). A thermogravimetric analysis (TGA) of the synthesized nanocomposites was performed on a Thermal Analysis Q500 instrument (TA Instruments, New Castle, DE, USA). Samples of about 2 mg were heated under nitrogen flow, with 10 °C/min, from 25 °C to 700 °C. The glass transition temperatures (T_g_) of the polyurea-polyurethane nanocomposites were established using differential scanning calorimetry (DSC). All the samples of around 10 mg were analyzed using a NETZSCH DSC 204 F1 Phoenix instrument (NETZSCH, Selb, Germany), under nitrogen flow, at 10 °C/min heating rate, in two heating/cooling cycles, between −80 and 200 °C. Stress–strain curves were obtained using an Instron 3382 testing machine (Instron, Norwood, MA, USA). The samples were prepared for the tensile tests by cutting the nanocomposite films in a rectangular shape, at standard dimensions for tensile specimen, with 5 mm width and 100 mm length. For each specimen, the rate of the extension was set at 500 mm/min, and the separation of the initial jaws was set at 50 mm (plain jaw faces). For each type of nanocomposite film, five tensile tests were carried out and the average of the measured values and the standard deviation for each point was registered. For a more accurate approach of the interpretation of these tests, the values of true stress (σ_T_) and true strain (ε_T_) were employed for determining Young’s modulus values, which were calculated according to the mathematical model described in [32]. The definition of true stress (σ_T_) states that this σ_T_ signifies the instantaneous applied load divided by the instantaneous cross-sectional area. True stress is related to engineering stress (σ) through the following equation: σ_T_ = σ (1 + ε). The definition of true strain (ε_T_) states that ε_T_ signifies the rate of instantaneous increase in the instantaneous gauge length. The true strain is related to engineering strain (ε) by ε_T_ = ln (1 + ε). A comparative multigraph containing all the true stress/true strain values characteristic for each synthesized material was plotted to evaluate the influence of the nanofiller on their mechanical properties. This multigraph was designed to show only the curves with the closest parameters to the mean values from each set of specimens. The dynamic and mechanical behavior of the samples was evaluated in single cantilever bending mode between −80 and 200 °C, with a controlled heating rate of 5 °C/min, using a TRITEC 2000 B-Dynamic mechanical analysis (DMA, Martignat, France) instrument.

For the evaluation of the behavior of polyurea-polyurethane nanocomposite films in a dynamic regime, a series of experimental measurements were performed on aluminum metal plates coated with polyurea and polyurea-polyurethane nanocomposites. The measuring instruments used were an ultra-high-speed video camera PHOTRON (Photron, Tokyo, Japan) and a PCB force transducer (PCB Piezotronics, Depew, NY, USA).

**Figure 1:**
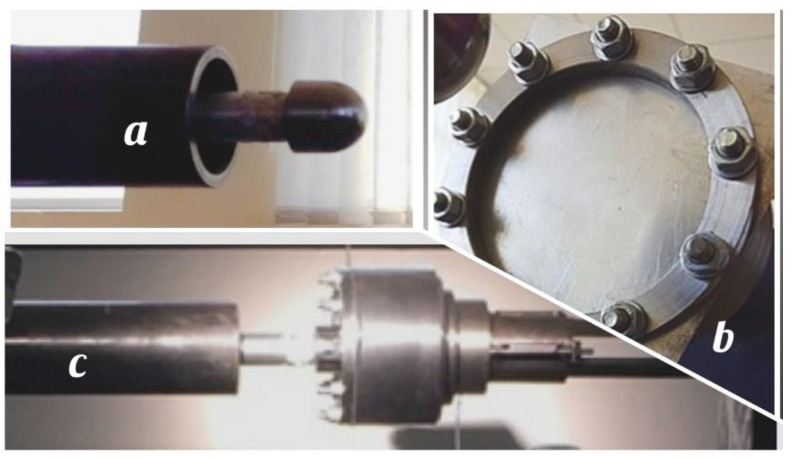
Hopkinson bar set-up for experimental testing of the polyurea-polyurethane-MWCNTs nanocomposite-coated aluminum plates in dynamic regime: (**a**) Spherical head projectile; (**b**) sample before the experiment (coated on the backside with polyurea (PU) or polyurea-polyurethane-MWCNTs nanocomposite (PU-NC)); (**c**) image captured during the experiment, at the moment of the impact of the projectile with the sample.

The samples used for these tests consisted of an aluminum metal plate with a thickness of 0.5 mm and a free diameter of 100 mm, on which a layer of polyurea-polyurethane nanocomposite of approximately 1 mm was previously deposited. Each sample was fixed (Figure 1b) and orientated, with the uncoated side towards the projectile launching direction. The experiments were carried out using a Hopkinson bar air propulsion system (Figure 1c), utilizing a spherical head projectile (Figure 1a). The impact strength obtained at the impact between the projectile and the samples was measured using a piezoelectric sensor connected to the mounting bracket of the tested aluminum plate. These experiments were performed on three types of materials: neat aluminum plate, polyurea coated aluminum plate (PU), and polyurea-polyurethane-MWCNTs nanocomposite-coated aluminum plates (PU-NC2, PU-NC3, and PU-NC4).

## 3. Results and Discussion

Polyurea and MWCNT nanocomposites designed for ballistic protection applications must meet certain performance criteria related to their thermal and mechanical strength. Materials exposed to the impact with a projectile or at the action of a shock wave can suffer both considerable deformation (or failure of the structure) and thermal degradation. Thus, polyurea coatings have the role of reducing these devastating effects.

**Figure 2:**
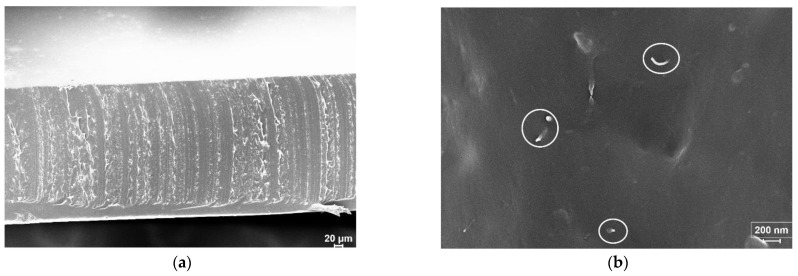
SEM analysis of the polyurea-polyurethane-MWCNTs nanocomposite films: (**a**) in cross-section; (**b**) from the top.

The first step in our study consisted in the morphology characterization of the nanocomposites using SEM analysis. In Figure 2 SEM images of the PU-NC4 sample are presented. The nanocomposite consists of a continuous film; however, it can be noticed that, in some areas (highlighted with a white ring), there is a lack of homogeneity. The explanation could be due to the MWCNTs’ aggregation in the polymer matrices and their escape at the surface of the polymer films, respectively.

**Figure 3:**
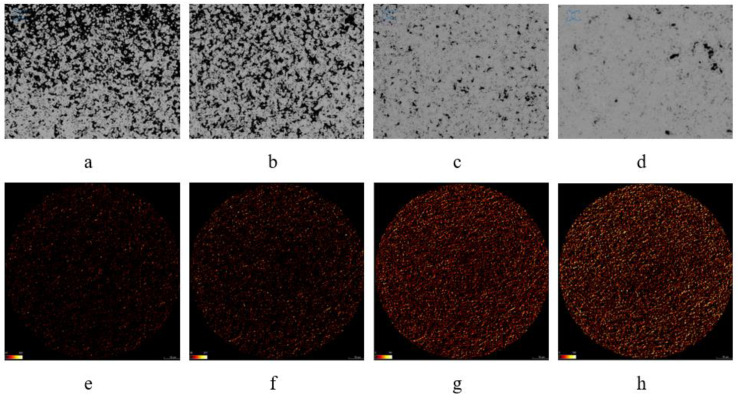
Micro-CT images (70 µm voxel size) of polyurea-polyurethane-MWCNTs nanocomposites comprising: (**a**,**e**) 0.05 wt.% MWCNTs, (**b**,**f**) 0.1 wt.% MWCNTs, (**c**,**g**) 0.2 wt.% MWCNTs, (**d**,**h**) 0.3 wt.% MWCNTs.

**Figure 4:**
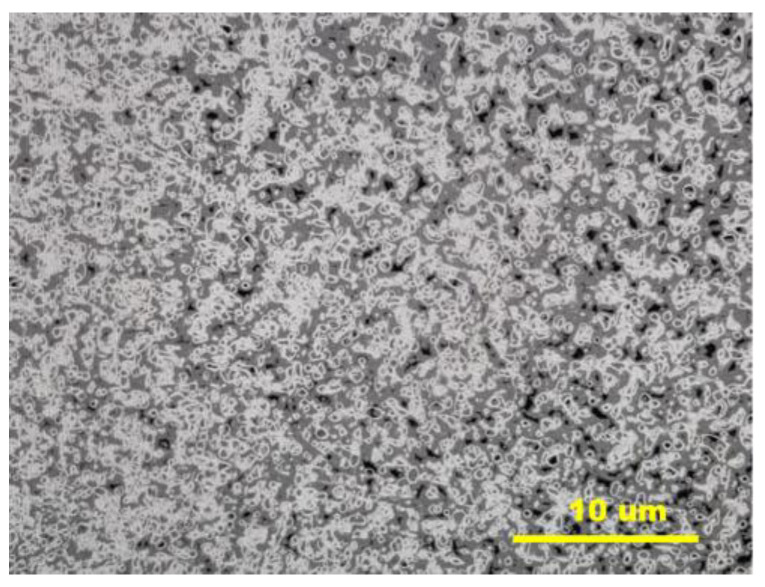
Micro-CT image (10 µm voxel size) of PU-NC4 sample.

To strengthen the hypothesis about the aggregation of the MWCNTs in sample PU-NC4, the second step of this study involved an investigation of the distribution of the MWCNTs inside the polyurea-polyurethane matrix through a micro-CT technique. CTVol^®^ Micro-CT Surface Rendering Software allowed a realistic visualization of the samples (Figure 3a–d and Figure 4) and displayed a homogenous spatial distribution of the nanofiller inside the polymeric matrix. Reconstruction of the collected images was performed by the NRecon reconstruction software (local version), and the full set of reconstruction results were visualized via DataViewer^®^ 2D/3D Micro-CT Slice Visualization(Micro Photonics Inc., Allentown, PA, USA). Representative 2D cross-section slices of each reconstructed sample were illustrated in Figure 3e–h. As can be observed from Figure 3, the MWCNTs are well dispersed inside the polyurea-polyurethane matrix. Moreover, the increase of MWCNTs concentration is well distinguishable in the four micro-tomographs.

Even if the nanocomposite samples containing 0.3 wt.% MWCNTs (PU-NC4) exhibited an apparent macro-homogeneity, there are regions where these nanoparticles tend to agglomerate, as can be observed in Figure 4.

**Figure 5:**
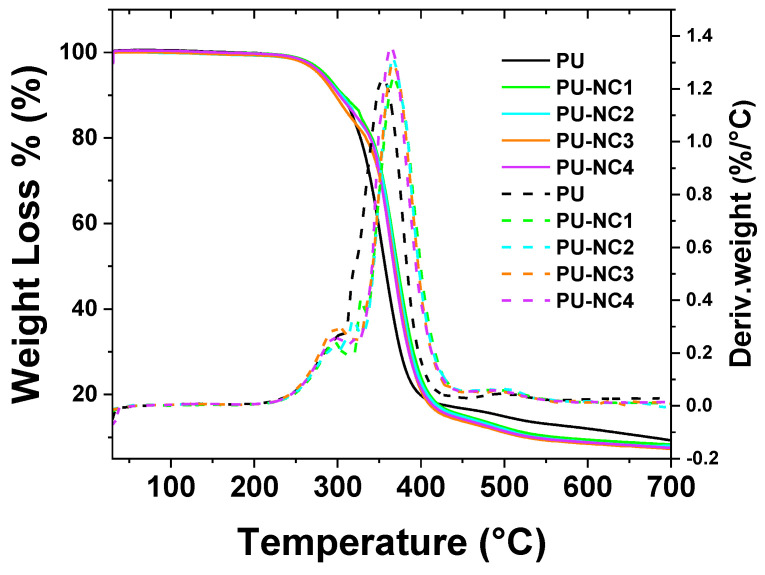
TGA and DTG analyses of the polyurea-polyurethane-MWCNTs nanocomposites.

Thermogravimetric analysis showed that the synthesized polyurea-polyurethane-MWCNTs films displayed a high thermal resistance, as can be observed in Figure 5 and Table 2 and Table 3.

According to the TGA analysis, it appears that the introduction of carbon nanotubes into polyurea films had a slightly positive effect on thermal resistance, as their degradation process began later compared to the control sample (PU). However, as the concentration of carbon nanotubes increased, this effect decreased. The temperature corresponding to the maximum decomposition rate was also higher in the case of nanocomposites. Starting at 354 °C (in the case of PU samples) and at 366 °C, respectively (in the case of the nanocomposite samples), a significant weight loss process could be observed due to the decomposition process of the polymeric matrix (Figure 5).

**Figure 6:**
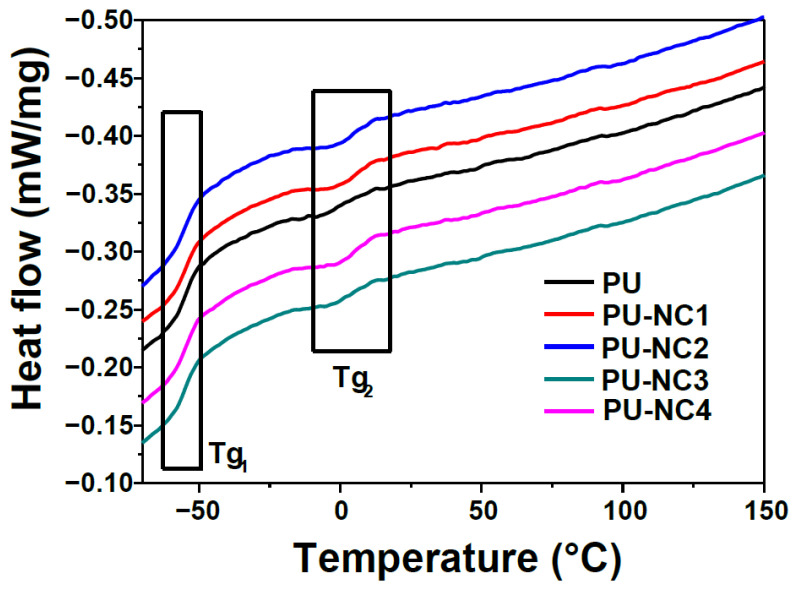
DSC curves obtained for polyurea-polyurethane-MWCNTs nanocomposites.

Another important aspect related to the performance of polyurea-based materials designed for ballistic protection is related to their glass transition temperature. At lower temperatures, but still above the value of the glass transition temperature, polyurea tends to pass into the glass phase due to the energy dissipation phenomenon generated during a deformation caused by the action of a shock wave or impact with a projectile [17,20,31]. As can be observed in Figure 6, the synthesized materials exhibited two glass transition temperatures, each of them corresponding to the flexible and rigid nanodomains of the polyurea nanocomposite, respectively. Table 4 summarizes the two T_g_ values obtained for the synthesized materials. It can be noticed that the values obtained for the glass transition temperatures associated with the flexible nanodomains (T_g1_) were quite similar to the blank sample (PU) because there are no significant differences between the aliphatic chains situated at a considerable distance from the rigid nanodomains, but there is a visible difference between the T_g2_ values obtained for the reference sample (PU) and the nanocomposite samples (PU-NC) because the carbon nanofiller joins the rigid nanodomains, thus reducing the mobility of the polymeric chains situated in their proximity.

**Figure 7:**
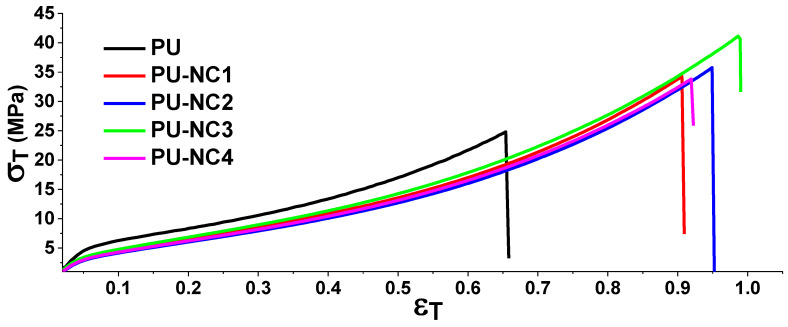
True stress–true strain plots for the synthesized polyurea-polyurethane nanocomposite films.

To evaluate the behavior of polyurea-polyurethane films under the mechanical stress action, the samples were subjected to tensile tests. Table 5 and Figure 7 illustrate the obtained results. As can be noticed, the introduction of carbon nanotubes into the polyurea matrix led to an increase of Young’s modulus, which means that they have become more rigid than the reference sample.

Although these materials were stiffer than the control sample, they showed higher deformation energy, which suggests that these materials can dissipate more energy. Since these materials were specially designed for ballistic protection, their high capacity to absorb and dissipate energy represents a remarkable advantage.

**Figure 8:**
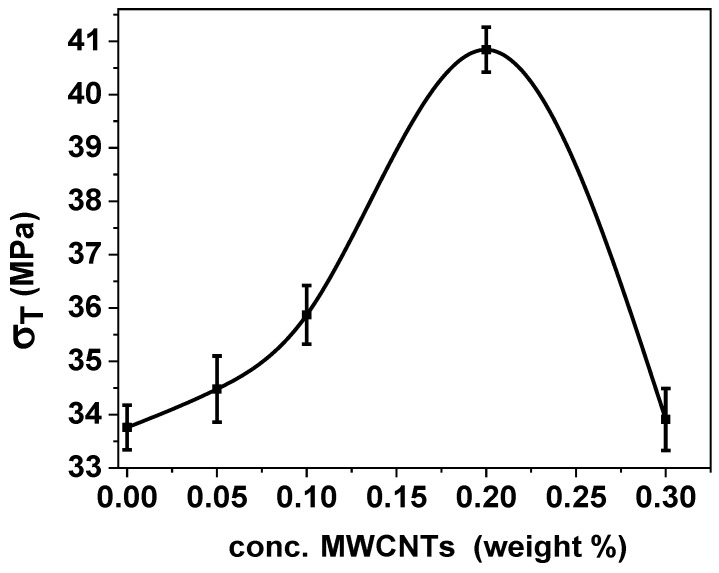
Variation of the maximum of true stress, depending on the concentration of the nanofiller.

The pre-dispersed MWCNTs achieved a good dispersion in the polymer matrix until it reached a concentration of 0.2% MWCNTs but, at higher concentrations, the nanofiller tends to agglomerate. This situation leads to a decrease in the tensile strength of the samples containing 0.3% MWCNTs (PU-NC4). Since PU-NC3 displayed the highest value of tensile true strain (σ_T_) and the highest deformation energy (Figure 7 and Table 5), we can affirm that these maximum values indicate the concentration of 0.2% MWCNTs as representing the optimal composition (Figure 8) for this type of polyurea nanocomposite. The results are also sustained by SEM and Micro-CT analyses through the aggregation of the MWCNTs in the case of PU-NC4.

**Figure 9:**
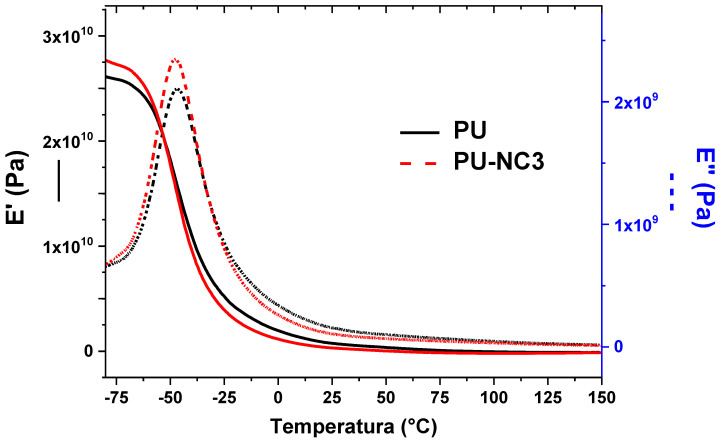
Comparative E′ and E″ plot between polyurea and polyurea-polyurethane nanocomposites.

The impact strength of a material is an index of its toughness. When a load is applied to a polymer, a part of the energy is dissipated throughout the polymer mass, while another part is stored in the material and will be released after the load is removed. The DMA analysis aimed to follow the evolution of storage (E′) and loss (E″) modulus of the synthesized nanocomposites to evaluate their potential to be used as coatings for the structures that need improved ballistic protection. Figure 9 illustrates a comparative plot of the E′ and E″ values for the blank sample (PU), and for the nanocomposite which displayed the best tensile test results (PU-NC3).

Both E′ and E″ were higher in the case of PU-NC3, showing that this nanocomposite possesses the ability to store a significant amount of energy, and it can also dissipate a higher quantity of energy in comparison with the neat polyurea (PU). The peaks corresponding to the loss modulus can allow the estimation of the glass transition temperature at around −50 °C, indicating that this value obtained through DMA technique is following DSC analysis. 

The characterization of these materials specially designed for ballistic protection would not be complete without experimental measurements which aim to evaluate their behavior at the impact with a projectile or the action of a shock wave. Therefore, for evaluating the efficiency of the obtained materials, round-headed projectiles were employed for impacting aluminum plates coated with the synthesized polyurea-polyurethane nanocomposites on the backside.

**Figure 10:**
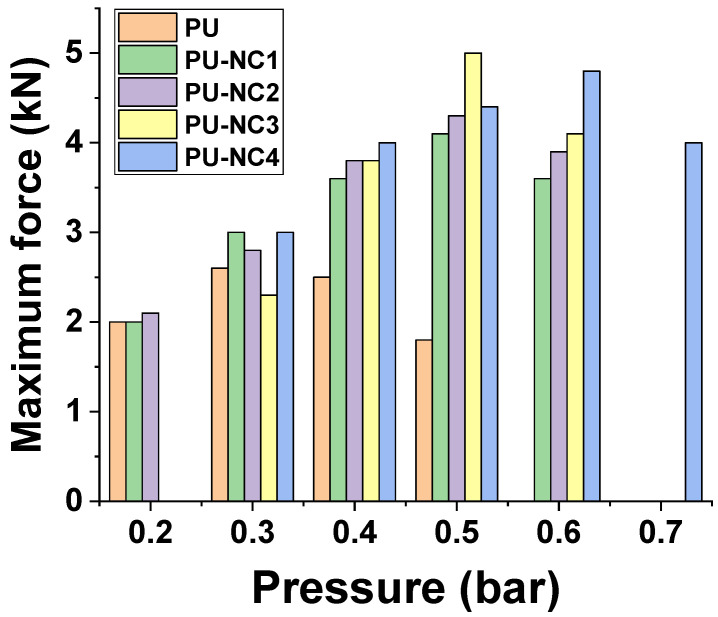
Maximum force values obtained during the impact tests with Hopkinson bar.

For the experimental testing in the dynamic regime, we used a Hopkinson bar set-up, with every sample being fixed and aligned for axial symmetry. An air gun, which allows the variation of pressure, launches the striker (the spherical head projectile in our case) towards the direction of the center of the tested specimen. Thus, the sample is rapidly impacted by the compressive stress wave generated. At impact, the samples undergo rapid and permanent deformations or fractures. Table 6 and Table 7 and Figure 10 display the results obtained with the Hopkinson bar tests. As can be noticed in Table 7, at an initial projectile acceleration pressure of 0.2 bars, all the samples underwent deformations, but none of them suffered fissures. Starting with 0.3 bars, small cracks appeared in the aluminum plate, but the samples covered with polyurea, even if they displayed higher deformations, did not suffer fractures, meaning that neat polyurea already offers significant improvement. At a higher initial projectile acceleration pressure of 0.4 bars, the aluminum plate underwent a higher fracture, but the polyurea-coated specimens did not exhibit fissures. Only at 0.5 bars of initial projectile acceleration pressure could a small fissure be observed on the aluminum plate coated with polyurea, but the polyurea film remained unbroken due to its higher elasticity, and the specimens coated with the nanocomposite suffered only low deformations and no fractures. At a higher initial projectile acceleration pressure of 0.6 bars, polyurea films PU and the nanocomposite containing the lowest MWNCTs concentration PU-NC2 started to crack. At 0.6 bars, the aluminum plate coated with PU-NC3 suffered low fractures, but this polymeric film resisted up to 0.8 bars. Therefore, we can affirm that, only at values higher than 0.8 bars for the initial projectile acceleration pressure, did both layers (metallic layer and polymeric nanocomposite coating) undergo visible fractures; at lower values, the metallic layer was the only one that cracked. In the case of PU-NC4 specimens, the metallic layer exhibited low fissures at 0.7 bars, and both layers (metallic plate and polymeric nanocomposite coating) were fractured at values higher than 0.8 bars. The increase in the resistance of the aluminum plates covered with PU and PU-NC can also be highlighted by the recordings made with the help of the pressure transducer (Table 6). From the values displayed in Table 6, it can be noticed that, for the tests performed on the uncoated metallic plates, the maximum value of the force was 2.6 kN at the pressure of 0.3 bar. This characteristic displayed a decreasing trend for the tests performed at higher pressures, and could be associated with the loss of the integrity of the plate (fracturing phenomenon). On the other hand, for the specimens coated with polyurea, the values measured with the pressure transducer were almost double for the maximum force, more precisely 5 kN for PU-NC2 at 0.5 bars. For higher initial projectile acceleration pressure, all specimens, including the ones coated with the nanocomposite, followed the same trend: after reaching a maximum value of the force, there was a decrease, a phenomenon associated with the loss of the integrity of the metal plates.

## 4. Conclusions

Polyurea-polyurethane and MWCNTs nanocomposites were obtained to be used for ballistic protection applications through a facile synthesis approach involving MWCNTs pre-dispersed in a polyester polyol-based resin. To demonstrate that these materials are suitable for this type of application, they were subjected to thermal and mechanical characterization using different analysis techniques (SEM, µCT, TGA, DSC, DMA, tensile tests), and they were also subjected to ballistic tests using a Hopkinson bar system. SEM and micro-CT analyses confirmed the homogenous dispersion of the MWCNTs inside the polyurea-polyurethane matrix for the samples containing 0.05%, 0.1%, and 0.2% MWCNTs. In the nanocomposites containing 0.3% MWCNTs, it can be noticed that, in some regions, there is a lack of homogeneity due to the tendency to form aggregates at higher concentrations of this nanofiller. TGA showed that the nanocomposite films have good thermal stability (up to about 300 °C). The presence of MWCNTs delayed the onset of the decomposition process of polyurea-polyurethane films, and the maximum degradation temperature was about 10 °C higher than that of the reference polyurea sample (PU). DSC curves displayed two glass transition temperatures due to the coexistence of the two segregated nanodomains from the componence of polyurea matrix (flexible and rigid nanodomains). According to tensile test results, it turned out that, from all the synthesized materials, the polyurea-polyurethane nanocomposite film containing 0.2% MWCNTs is the optimal option for ballistic protection applications, since it possesses the highest deformation energy. DMA analysis also demonstrated that PU-NC3 samples had a remarkable capacity for absorbing and dissipating energy. Experimental testing in a dynamic regime of the polyurea-coated aluminum plates showed that the polymeric layer allows the metal plate to maintain its integrity at an acceleration pressure value that is almost three times higher than the one for the uncoated metallic specimen. Synthesized nanocomposites possess unique properties that recommend them to be used in the modernization of ballistic protection equipment and devices. Thus, military vehicles or bulletproof vests could be safer, but also lighter and less expensive. 

We can conclude that the synthesized polyurea-polyurethane-MWCNTs nanocomposites possessing this exceptional combination of properties and advantages are suitable for employment as complementary materials for ballistic protection.

## Figures and Tables

**Table 1:** The composition of polyurea nanocomposite films.

Sample	MWCNTs (wt. %)
PU (blank)	0
PU-NC1	0.05
PU-NC2	0.1
PU-NC3	0.2
PU-NC4	0.3

**Table 2:** Thermal properties of polyurea-polyurethane-MWCNTs nanocomposites.

Sample	T_10%_, [°C]	T_max_, [°C]
PU	303.0	354.0
PU-NC1	307.0	367.0
PU-NC2	304.5	366.5
PU-NC3	303.5	366.5
PU-NC4	302.0	364.0

T_10%_ = decomposition onset temperature (measured at 10% weight loss); T_max_ = the maximum decomposition temperature, corresponding to the maximum of DTG peak (the first derivative of the thermogravimetric curve).

**Table 3:** The decomposition process of the polyurea-polyurethane-MWCNTs nanocomposites.

Sample	Weight Loss [%]								
	200 °C	250 °C	300 °C	350 °C	400 °C	450 °C	500 °C	550 °C	600 °C
PU	0.2	1.3	9.1	41.4	80.2	83.3	85.1	86.9	87.9
PU-NC1	0.2	1.1	8.5	25.7	75.9	84.8	87.7	89.7	90.6
PU-NC2	0.7	1.7	9.0	26.2	77.1	85.2	88.1	90.2	91.1
PU-NC3	0.6	1.8	9.8	28.7	79.1	86.3	88.9	90.7	91.5
PU-NC4	0.2	1.3	9.4	28.1	78.5	85.7	88.6	90.4	91.2

**Table 4:** Glass transition temperature obtained for polyurea-polyurethane-MWCNTs nanocomposites (Tg1–glass transition corresponding to the flexible nanodomains; Tg2–glass transition corresponding to the hard nanodomains).

Sample	Tg1, °C	Tg2, °C
PU	−54.0	6.0
PU-NC1	−54.5	9.5
PU-NC2	−55.5	10.0
PU-NC3	−55.5	10.0
PU-NC4	−55.5	10.0

**Table 5:** Mechanical properties of polyurea-polyurethane- MWCNTs nanocomposites.

Sample	MWCNTs Conc. (%)	Young’s Modulus * (MPa)	Maximum Tensile True Stress (σ_T_) (MPa)	Deformation Energy (Tensile Toughness) (J·m^−3^)
PU	0	48.65 ± 1.02	33.76 ± 0.42	11.31 ± 0.29
PU-NC1	0.05	70.11 ± 0.98	34.48 ± 0.62	12.90 ± 0.13
PU-NC2	0.1	79.22 ± 1.28	35.87 ± 0.55	13.57 ± 0.21
PU-NC3	0.2	81.54 ± 0.83	40.84 ± 0.42	16.53 ± 0.18
PU-NC4	0.3	43.87 ± 1.44	33.91 ± 0.58	12.90 ± 0.43

* Young’s Modulus was calculated according to the mathematical model described by Xue and Hutchinson [32].

**Table 6:** Maximum force values obtained during the impact tests with Hopkinson bar.

Pressure (Bar)	Aluminum Plate (Uncoated)	Aluminum Plate (Coated with PU)	Aluminum Plate (Coated with PU-NC2)	Aluminum Plate (Coated with PU-NC3)	Aluminum Plate (Coated with PU-NC4)
	Maximum Force (kN)	Maximum Force (kN)	Maximum Force (kN)	Maximum Force (kN)	Maximum Force (kN)
0.2	2.0	2.0	2.1	-	-
0.3	2.6	3.0	2.8	2.3	3.0
0.4	2.5	3.6	3.8	3.8	4.0
0.5	1.8	4.1	4.3	5.0	4.4
0.6	-	3.6	3.9	4.1	4.8
0.7	-	-	-	-	4.0

**Table 7:** Images of the tested specimens, captured after the impact tests with Hopkinson bar (pressure in bar and pattern of deformation).

**Aluminum Plate**	**Aluminum Plate + PU**	**Aluminum Plate + PU-NC2**	***Experimental Set-Up—Hopkinson bar***	
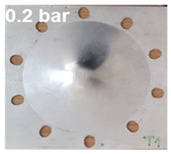	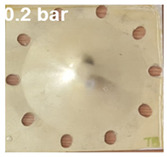	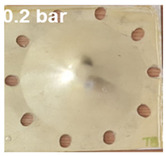	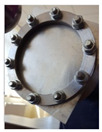	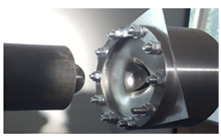
			**Aluminum Plate + PU-NC3**	**Aluminum Plate + PU-NC4**
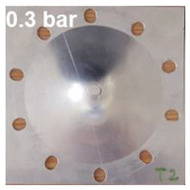	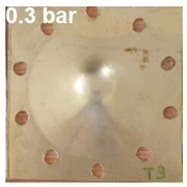	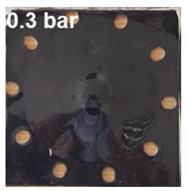	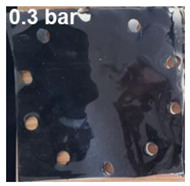	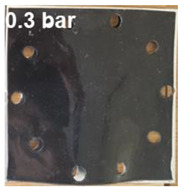
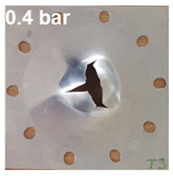	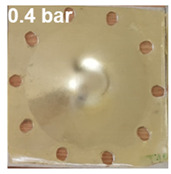	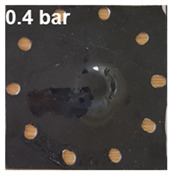	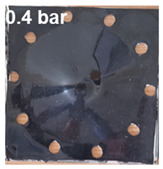	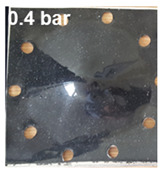
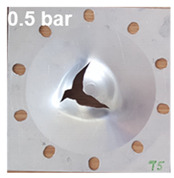	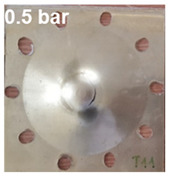	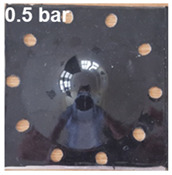	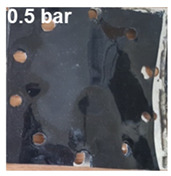	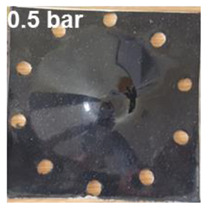
	**Aluminum Plate + PU-NC1**	**Aluminum Plate + PU-NC2**	**Aluminum Plate + PU-NC3**	**Aluminum Plate + PU-NC4**
	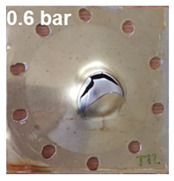	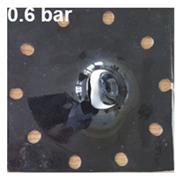	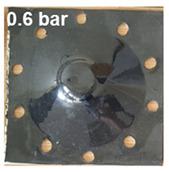	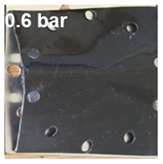
		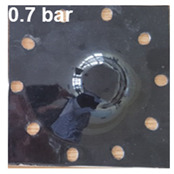	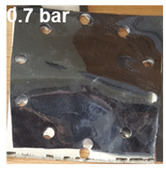	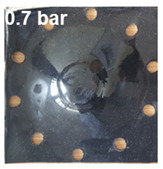
			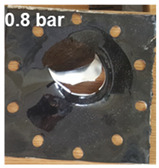	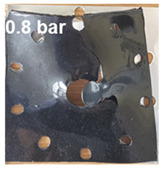

## References

[1] Parveen S., Rana S., Fangueiro R. (2013). A Review on Nanomaterial Dispersion, Microstructure, and Mechanical Properties of Carbon Nanotube and Nanofiber Reinforced Cementitious Composites. J. Nanomater..

[2] Goga D., Țigănescu T., Pulpea B., Moldoveanu C., Rotaru C. (2017). A Quantitative Method of Comparative Assessment of Primers Ignition Performances. Adv. Mil. Technol..

[3] Pulpea G.B., Voicu A., Matache L., Haller L., Mandache-Dodoiu A.D., Ginghină R. (2017). Numerical Simulation of Interior Ballistics for Pyrotechnics Systems. J. MTA Rev..

[4] Gavrila A.M., Iordache T.V., Lazau C., Rotariu T., Cernica I., Stroescu H., Stoica M., Orha C., Bandas C.E., Sarbu A. (2020). Biomimetic Sensitive Elements for 2,4,6-Trinitrotoluene Tested on Multi-Layered Sensors. Coatings.

[5] Voicu A.E., Rotariu T., Teodorescu M., Zecheru T., Tiganescu T.V., Orban O. (2017). pH sensitive polymeric binders for energetic materials. Mater. Plast..

[6] Da Silva J.E.L., Paciornik S., d’Almeida J.R.M. (2004). Evaluation of the effect of the ballistic damaged area on the residual impact strength and tensile stiffness of glass-fabric composite materials. Compos. Struct..

[7] Ávila A.F., Neto A.S., Nascimento H. (2011). Hybrid nanocomposites for mid-range ballistic protection. Int. J. Impact Eng..

[8] Findik F., Tarim N. (2003). Ballistic impact efficiency of polymer composites. Compos. Struct..

[9] Casalini R., Bogoslovov R., Qadri S.B., Roland C.M. (2012). Nanofiller reinforcement of elastomeric polyurea. Polymer.

[10] Bogue R. (2011). Nanocomposites: A review of technology and applications. Assem. Autom..

[11] Bhattacharya M. (2016). Polymer Nanocomposites—A Comparison between Carbon Nanotubes, Graphene, and Clay as Nanofillers. Materials.

[12] Ho M.-W., Lam C.-K., Lau K.-T., Ng D.H.L., Hui D. (2006). Mechanical properties of epoxy-based composites using nanoclays. Compos. Struct..

[13] Crosby A.J., Lee J.Y. (2007). Polymer Nanocomposites: The “Nano” Effect on Mechanical Properties. Polym. Rev..

[14] Hsiao C.-C., Lin T.S., Cheng L.Y., Ma C.-C.M., Yang A.C.M. (2005). The Nanomechanical Properties of Polystyrene Thin Films Embedded with Surface-grafted Multiwalled Carbon Nanotubes. Macromolecules.

[15] Kumar V., Lee G., Monika, Choi J., Lee D.-J. (2020). Studies on composites based on HTV and RTV silicone rubber and carbon nanotubes for sensors and actuators. Polymer.

[16] Kumar V., Alam M.N., Manikkavel A., Choi J., Lee D.-J. (2020). Investigation of silicone rubber composites reinforced with carbon nanotube, nanographite, their hybrid, and applications for flexible devices. J. Vinyl Addit. Technol..

[17] Toader G., Rusen E., Teodorescu M., Diacon A., Stanescu P.O., Rotariu T., Rotariu A. (2016). Novel polyurea polymers with enhanced mechanical properties. J. Appl. Polym. Sci..

[18] Raman S.N., Ngo T., Lu J., Mendis P. (2013). Experimental investigation on the tensile behavior of polyurea at high strain rates. Mater. Des..

[19] Mohotti D., Ngo T., Raman S.N., Ali M., Mendis P. (2014). Plastic deformation of polyurea coated composite aluminium plates subjected to low velocity impact. Mater. Des..

[20] Grujicic M., Pandurangan B., He T., Cheeseman B.A., Yen C.F., Randow C.L. (2010). Computational investigation of impact energy absorption capability of polyurea coatings via deformation-induced glass transition. Mater. Sci. Eng. A.

[21] Fragiadakis D., Gamache R., Bogoslovov R.B., Roland C.M. (2010). Segmental dynamics of polyurea: Effect of stoichiometry. Polymer.

[22] Grujicic M., He T., Pandurangan B., Svingala F.R., Settles G.S., Hargather M.J. (2012). Experimental Characterization and Material-Model Development for Microphase-Segregated Polyurea: An Overview. J. Mate. Eng. Perform..

[23] Ackland K., Anderson C., Ngo T.D. (2013). Deformation of polyurea-coated steel plates under localised blast loading. Int. J. Impact Eng..

[24] Li T., Zheng T., Han J., Liu Z., Guo Z.-X., Zhuang Z., Xu J., Guo B.-H. (2019). Effects of Diisocyanate Structure and Disulfide Chain Extender on Hard Segmental Packing and Self-Healing Property of Polyurea Elastomers. Polymers.

[25] Amini M.R., Isaacs J., Nemat-Nasser S. (2010). Investigation of effect of polyurea on response of steel plates to impulsive loads in direct pressure-pulse experiments. Mech. Mater..

[26] Roland C.M., Fragiadakis D., Gamache R.M., Casalini R. (2013). Factors influencing the ballistic impact resistance of elastomer-coated metal substrates. Philos. Mag..

[27] Bai Y., Liu C., Huang G., Li W., Feng S. (2016). A Hyper-Viscoelastic Constitutive Model for Polyurea under Uniaxial Compressive Loading. Polymers.

[28] Li Y., Chen C., Hou H., Cheng Y., Gao H., Zhang P., Liu T. (2019). The Influence of Spraying Strategy on the Dynamic Response of Polyurea-Coated Metal Plates to Localized Air Blast Loading: Experimental Investigations. Polymers.

[29] Petre R., Zecheru T., Petrea N., Ginghina R., Sandu S., Muresan M., Matache L.C., Sava A.C., Neatu F. (2018). Synthesis and Mechanical Properties of Polyurea-Based Hybrid Composites for Ballistic Individual Protection. Mater. Plast..

[30] Sahoo N.G., Rana S., Cho J.W., Li L., Chan S.H. (2010). Polymer nanocomposites based on functionalized carbon nanotubes. Prog. Polym. Sci..

[31] Toader G., Rusen E., Teodorescu M., Diacon A., Stanescu P.O., Damian C., Rotariu T., Rotariu A. (2017). New polyurea MWCNTs nanocomposite films with enhanced mechanical properties. J. Appl. Polym. Sci..

[32] Xue Z., Hutchinson J.W. (2007). Neck retardation and enhanced energy absorption in metal–elastomer bilayers. Mech. Mater..

